# Diagnostic Efficacy of Ultrasound, Cytology, and BRAF^V600E^ Mutation Analysis and Their Combined Use in Thyroid Nodule Screening for Papillary Thyroid Microcarcinoma

**DOI:** 10.3389/fonc.2021.746776

**Published:** 2022-01-03

**Authors:** Jing Du, Ruijun Han, Cui Chen, Xiaowei Ma, Yuling Shen, Jun Chen, Fenghua Li

**Affiliations:** ^1^ Department of Ultrasound, Renji Hospital, Shanghai Jiao Tong University School of Medicine, Shanghai, China; ^2^ Department of Laboratory Medicine, Renji Hospital, Shanghai Jiao Tong University School of Medicine, Shanghai, China; ^3^ Department of Head and Neck Surgery, Renji Hospital, Shanghai Jiao Tong University School of Medicine, Shanghai, China

**Keywords:** thyroid nodule, papillary thyroid microcarcinoma, ultrasound, fine-needle aspiration, BRAF^V600E^ mutation

## Abstract

**Background:**

Ultrasound, cytology, and BRAF^V600E^ mutation analysis were applied as valuable tools in the differential diagnosis of thyroid nodules. The aim of the present study was to evaluate the diagnostic efficiency of the three methods and their combined use in screening for papillary thyroid microcarcinoma (PTMC).

**Methods:**

A total of 1,081 patients with 1,157 thyroid nodules (0.5–1 cm in maximum diameter) classified as thyroid imaging reporting and data system (TIRADS) 4–5 were recruited. All patients underwent ultrasound, fine-needle aspiration (FNA) examination, and an additional BRAF^V600E^ mutation test. TIRADS and Bethesda System for Reporting Thyroid Cytopathology (BSRTC) were adopted to judge the ultrasound and cytological results. The receiver operating characteristic (ROC) curve was established to assess the diagnostic values of different methods.

**Results:**

Of the 1,157 nodules, 587 were benign and 570 were PTMCs. BRAF^V600E^ mutation test had highest sensitivity (85.4%), specificity (97.1%), accuracy (91.4%), and area under the ROC curve (Az) value (0.913) among the three methods. The combination of BSRTC and BRAF^V600E^ mutation analysis yielded a considerably high sensitivity (96.0%), accuracy (94.3%), and negative predictive value (95.9%) than either BSRTC or BRAF^V600E^ mutation alone (*P* < 0.0001 for all comparisons). Of all the methods, the combined use of the three methods produced the best diagnostic performance (Az = 0.967), which was significantly higher than that (Az = 0.943) for the combination of BSRTC and BRAF^V600E^ mutation (*P* < 0.0001). The diagnostic accuracy of the molecular method in the 121 nodules with indeterminate cytology was 90.1% (109/121), which was significantly higher than that of TIRADS classification, 74.4% (90/121) (*P* = 0.002).

**Conclusion:**

The combined use of ultrasound, cytology, and BRAF^V600E^ mutation analysis is the most efficient and objective method for diagnosing PTMC. Both BRAF^V600E^ mutation and TIRADS classification are potentially useful adjuncts to differentiate thyroid nodules, especially indeterminate samples classified as BSRTC III.

## Introduction

Papillary thyroid microcarcinoma (PTMC) is defined as papillary thyroid carcinoma (PTC) that is less than 1 cm in its greatest diameter (1). It is the most common form of thyroid cancer, accounting for approximately half of the increased incidence in PTC (2). The early clinical recognition of PTMC is important due to high risk of lymph node metastasis and multicentricity. According to the previous reports, lymph node metastasis is highly prevalent in PTMC, resulting in a poor prognosis (3–5).

Ultrasound is the most sensitive modality available to detect thyroid nodules and useful in selecting the high-risk lesions for fine-needle aspiration (FNA) by detecting ultrasonographic features suggestive of malignancy. Thyroid imaging reporting and data system (TIRADS) was carried out and utilized by radiologists worldwide on the basis of ultrasonographic imaging features to stratify malignant risks of thyroid nodules (6). The diagnosis of PTMC relies on ultrasound examinations, whereas the lesions are difficult to assess and easily misdiagnosed due to their small size and atypical ultrasonographic features (7, 8).

Ultrasound-guided FNA is a routine and reliable approach for preoperative evaluation of thyroid nodules (9). Bethesda System for Reporting Thyroid Cytopathology (BSRTC) is developed to provide uniform terminology and diagnostic criteria, which has proven to be an effective and robust thyroid FNA classification scheme to guide the clinical management of patients with thyroid nodules (10). Despite the high sensitivity and specificity of thyroid cytology, the highlighted limitation is that about 30% of FNA cytologic findings remain non-diagnostic or indeterminate and may complicate the management of thyroid nodules (11). The prevalence of malignant tumors among these lesions varies from 10% to 52%, being mostly represented by PTCs that are diagnosed postoperatively (12).

A number of genetic analyses have been found to enhance the diagnostic accuracy of cytology and as a predictor of poor prognosis in patients with thyroid carcinomas. The most promising of these is the application of BRAF^V600E^ mutation analysis as a molecular marker for the detection of PTC. Numerous researchers have proven that the detection of BRAF^V600E^ mutation in FNA specimens refines the FNA-cytology diagnosis, especially in a BRAF^V600E^ mutation-prevalent area (13, 14), while others believe that its utility is limited by low prevalence of BRAF^V600E^ mutation in indeterminate nodules (15–17). Further studies are needed to identify which thyroid nodules should be rescreened using molecular tests.

The positive detection rate of BRAF^V600E^ mutation is associated with tumor size (18, 19). Moreover, the size of thyroid lesions might also have an impact on the diagnostic efficiency of ultrasound and FNA. To overcome and supplement the individual shortcomings of conventional ultrasound, ultrasound-guided FNA, and BRAF gene mutation analysis, several previous studies have tried to predict the probability of PTC by combining them (15, 20, 21), but none has focused on PTMC.

The purpose of this study, therefore, was to compare the diagnostic efficacy of ultrasound, cytology, and BRAF^V600E^ mutation analysis in the detection of PTMC and to explore the combined use of these methods for better diagnostic performance, especially focusing our attention on indeterminate thyroid nodules classified as BSRTC category III.

## Materials and Methods

### Study Population

**Figure 1:**
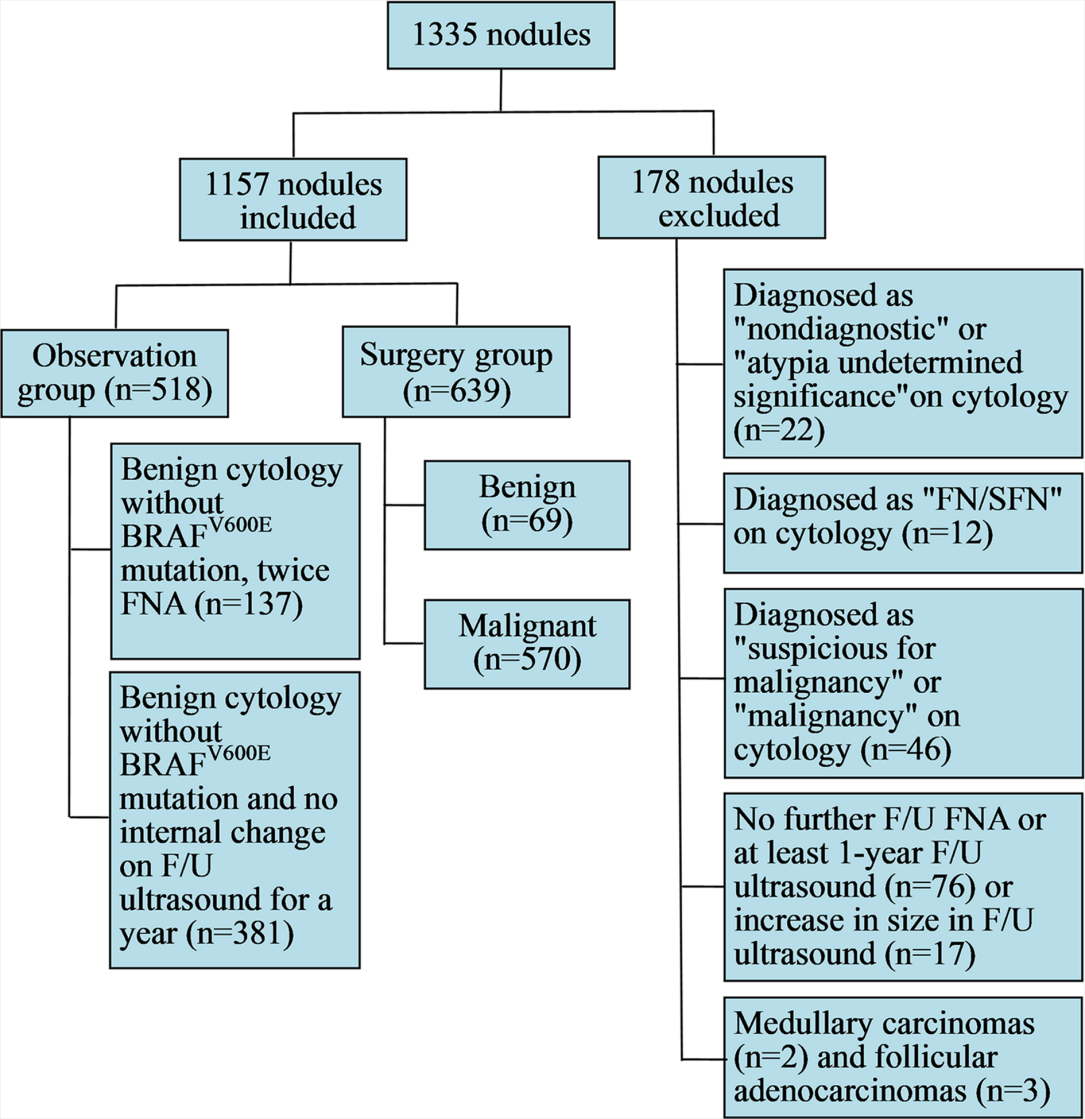
Diagram of the study group.

This prospective study was approved by the Ethics Committee of Renji Hospital, and written informed consent was obtained from each patient for ultrasound-guided FNA and BRAF^V600E^ mutation analysis prior to each procedure. This investigation was carried out following the rules of the Declaration of Helsinki. Between April 2019 and September 2020, 1,251 consecutive patients with 1,335 nodules (TIRADS categories 4 and 5, measured 5–10 mm in maximum diameter) underwent ultrasound and ultrasound-guided FNA with an additional BRAF^V600E^ mutation test. Of the 1,251 patients, only patients who met the following criteria were included: those who underwent surgery after thyroid ultrasound and FNA, those who underwent FNA at least twice with a 1-year interval for a benign thyroid lesion, and those who underwent FNA and follow-up (F/U) ultrasound (>12 months after FNA cytology diagnosis) for a benign thyroid lesion. All the patients with malignant lesions underwent thyroid lobectomy or total thyroidectomy with unilateral or bilateral prophylactic central neck dissection. Among the 1,251 patients with 1,335 nodules, 80 nodules in 79 patients were excluded because they showed cytologic results of non-diagnostic (n = 4), atypia undetermined significance/follicular lesion of undetermined significance (AUS/FLUS) (n = 18), follicular neoplasm or suspicious for a follicular neoplasm (FN/SFN) (n = 12), suspicious for malignancy (n = 27) and malignancy (n = 19) without further histopathological diagnosis or FNA and ultrasound F/U. Among the 478 nodules with benign cytologic results and negative BRAF gene mutation, 76 nodules in 69 patients were excluded because they did not undergo F/U FNA or at least 1-year F/U ultrasound, and another 17 nodules in 17 patients were excluded because they showed an increase in size in F/U ultrasound without further cytologic or hispathologic evaluation. Other types of thyroid malignancies were also excluded including two medullary thyroid carcinomas and three follicular adenocarcinomas. Finally, 1,081 patients with 1,157 thyroid nodules were included in this study. The mean maximum diameter of the nodules was 7.6 ± 2.3 mm (range, 5.2–9.9 mm). Among the 1,157 nodules, 639 nodules were confirmed by operation (Surgery group) and the other 518 nodules were observed by F/U FNA with an additional BRAF^V600E^ mutation test or F/U ultrasound after a year (Observation group) (**Figure 1**). Patient characteristics including age, sex, multifocality, lymph node metastasis, association with Hashimoto’s thyroiditis, and family history of thyroid cancer were also recorded.

### Ultrasound Examination

Conventional ultrasound examination and measurement were performed by an ultrasound radiologist (JD) with 14 years of experience in thyroid ultrasound. A Philips EPIQ 7 scanner (Philips Medical Systems, Bothell, WA, USA) equipped with a 3- to 12-MHz linear-array transducer was used for careful ultrasound examination of the thyroid gland and neck region. The ultrasound criteria that we used in this study for the evaluation of solid and partially cystic thyroid nodules were based on a number of previous studies (22–24). Suspicious ultrasound features of thyroid malignancy were as follows: (a) solid component, hypoechogenicity or marked hypoechogenicity; (b) irregular margins or perinodular thyroid parenchyma invasion; (c) calcifications; (d) taller-than-wide shape; (e) partially cystic nodule with eccentric location of the fluid portion and lobulation of the solid component; (f) intranodular hypervascularization and peripherally penetrating vessels; (g) irregular thick halo; and (h) cervical lymphadenopathy with intranodal cystic components or microcalcifications.

According to TIRADS proposed by Kwak et al. (23) and Sánchez (24), all nodules were classified into four categories: 4a (one suspicious ultrasound feature), 4b (two suspicious ultrasound features), 4c (three or four suspicious ultrasound features), and 5 (five suspicious ultrasound features). Two radiologists (RH and FL, with 11 and 31 years of experience in thyroid ultrasound, respectively) did all the classifications. Every investigator clarified the reasons why they made the classifications, and a consensus was reached in cases of discrepancy. The nodules with TIRADS 4c and 5 were classified as malignant lesions, while those with TIRADS 4a and 4b were considered benign lesions.

### Ultrasound-Guided Fine-Needle Aspiration and Cytological Analysis

Ultrasound-guided FNA with an additional BRAF^V600E^ mutation analysis was performed by an experienced radiologist (JD) using a standardized protocol (20). At our institution, ultrasound-guided FNA was performed in the thyroid nodule with most suspicious ultrasound features. If a patient had multiple suspicious nodules, the one in the same lobe showing the highest risk of malignancy or two nodules in the bilateral thyroid lobes were selected for ultrasound-guided FNA.

Based on BSRTC published by Cibas and Ali (25), FNA cytology results were categorized by two experienced pathologists as I–VI: non-diagnostic, benign, AUS/FLUS, FN/SFN, suspicious for malignancy, and malignancy. BSRTC I–IV were considered benign, while BSRTC V–VI were considered malignant.

### DNA Isolation and BRAF^V600E^ Mutation Test

BRAF^V600E^ mutation detection was performed using a China Food and Drug Administration (CFDA)-approved human BRAF gene V600E mutation fluorescence polymerase chain reaction (PCR) diagnostic kit (AmoyDX, Xiamen, China). The quantity of isolated DNA was assessed by using a NanoDrop2000 spectrophotometer (Thermo, Los Angeles, CA, USA). The samples were analyzed by applying the amplification refractory mutation system (ARMS) technique (20).

### Criteria of the Combined Use of Different Methods

When two diagnostic methods combined, a nodule was considered positive as long as either method was positive. In our study, BRAF^V600E^ mutation showed the highest diagnostic performance for discrimination between benign and malignant thyroid lesions. Cytology or ultrasound alone was not enough to make a definite diagnosis when BRAF^V600E^ mutation was negative. Therefore, the following criteria were included in the combined use of the three methods: (a) when BRAF^V600E^ mutation was positive, thyroid nodules were considered malignant tumors regardless of cytology and ultrasound; (b) when BRAF^V600E^ mutation was negative, thyroid nodules were considered malignant tumors if the results of both cytology and ultrasound were highly suggestive of malignancy; and (c) when BRAF^V600E^ mutation was negative, thyroid nodules were considered an uncertain diagnosis and suggestive of benign possibility if cytology or ultrasound alone was suspicious for malignancy.

### Statistical Analysis

The Student’s t-test and Mann–Whitney test were used to analyze numeric variables. Differences in the distribution of categorical variables between groups were evaluated by using a chi-square test. The McNemar chi-square test was used to compare the diagnostic sensitivity, specificity, and accuracy. The receiver operating characteristic (ROC) curve analysis was performed by using MedCalc V.16.4 statistical software (MedCalc Software, Ostend, Belgium). SPSS software (version 13.0, SPSS) was used for the statistical analysis, and a *P* value of < 0.05 was defined as statistical significance.

## Results

### Patients’ Findings

The 1,081 patients (815 women, 266 men; mean age, 48.8 years; range, 16–79 years) with 1,157 thyroid nodules were included in this study. There were 554 (51.2%) cases of benign nodules and 527 (48.8%) cases of malignancy. Of the 1,157 nodules, 587 were benign and 570 were malignant. All the diagnoses of malignant lesions were PTMCs. Diagnoses of benign lesions included nodular goiter (n = 376), Hashimoto’s thyroiditis (n = 28), adenoma (n = 102), subacute thyroiditis (n = 49), absorption phase cyst (n = 31), and follicular epithelial dysplasia (n = 1).

Detailed information on demographic and clinicopathologic characteristics of all the subjects enrolled in this study was summarized in **Table 1**. Patients with benign nodules were older than those with malignant nodules (mean age, 51.4 years ± 13.1 *vs*. 46.1 years ± 12.6, respectively; *P* < 0.0001). There was no statistically significant relationship between the risk of malignancy and sex or size (*P* = 0.452 and *P* = 0.741, respectively). Tumor multifocality was observed in 43 (8.2%) cases, with PTMCs and lymph node metastases in 150 (28.5%) patients. A total of 112 (21.3%) cases with PTMCs were combined with Hashimoto’s thyroiditis, and 21 (4.0%) had familial genetic history.

**Table 1:** Demographic and clinicopathologic characteristics of the study population.

Characteristics	Benign (n = 554)	Malignant (n = 527)	*P* value
Sex			
Male	131 (23.6)	135 (25.6)	0.452
Female	423 (76.4)	392 (74.4)	
Age (years)	51.4 ± 13.1	46.1 ± 12.6	<0.0001
Tumor size (mm)	7.6 ± 2.2	7.7 ± 2.3	0.741
Multifocality, no. (%) of nodules	–	43 (8.2)	–
Lymph node metastasis, no. (%) of nodules	–	150 (28.5)	–
Hashimoto’s thyroiditis, no. (%) of nodules	36 (6.5)	112 (21.3)	<0.0001
Familial genetic history, no. (%) of nodules	–	21 (4.0)	–

### Diagnostic Value of TIRADS

A total of 1,157 thyroid nodules were prospectively classified as 4a (n = 322), 4b (n = 354), 4c (n *=* 439), and 5 (n = 42). The malignancy risk increased as the level of TIRADS classification increased. The malignancy risks of categories 4a, 4b, 4c, and 5 were 14.6%, 36.4%, 80.2%, and 100%, respectively. The ROC curve demonstrated that the optimal cutoff point of TIRADS was 4c. The Az value, sensitivity, specificity, accuracy, positive predictive value (PPV), and negative predictive value (NPV) were 0.814, 69.1%, 85.2%, 77.3%, 81.9%, and 74.0%, respectively.

### Correlations of TIRADS Classification With BSRTC and BRAF^V600E^ Mutation

As shown in **Table 2**, the detection rates of BSRTC and BRAF^V600E^ mutation for PTMCs increased as the level of TIRADS classification increased. In nodules classified as TIRADS 4a, 4b, 4c, and 5, the detection rates of BRAF^V600E^ mutation were higher than those of BSRTC, whereas a statistical difference was found in only nodules classified as TIRADS 4c (*P* = 0.016), and there was no statistical difference in nodules classified as TIRADS 4a, 4b, and 5 (72.3% *vs*. 78.7%, *P* = 0.629; 74.4% *vs*. 83.7%, *P* = 0.088; 83.3% *vs*. 85.7%, *P* = 1, respectively). The combination of BSRTC and BRAF^V600E^ mutation significantly increased the detection rates for PTMCs with various levels of TIRADS classification compared with either BSRTC or BRAF^V600E^ mutation alone, and all the detection rates for PTMCs were above 90%. However, in nodules classified as TIRADS 5, no statistical difference was found between the combination of two methods and BSRTC or BRAF^V600E^ mutation alone (*P* = 0.063 and *P* = 0.125, respectively). This result indicated that when the nodule was classified as TIRADS category 5, a single detection method could be used to make a definite diagnosis without the need for a combination test.

**Table 2:** Correlations of TIRADS classification with detection rates of BSRTC and BRAF^V600E^ mutation for PTMCs.

TIRADS classification		n (%)	Malignancy (%)	BSRTC (%)	BRAF^V600E^ mutation (%)	BSRTC+ BRAF^V600E^ mutation (%)
4a	322 (27.8)		47 (14.6)	34 (72.3)	37 (78.7)	44 (93.6)
4b	354 (30.6)		129 (36.4)	96 (74.4)	108 (83.7)	123 (95.3)
4c	439 (37.9)		352 (80.2)	282 (80.1)	306 (86.9)	340 (96.6)
5	42 (3.6)		42 (100.0)	35 (83.3)	36 (85.7)	40 (95.2)
Total	1,157 (100)		570 (49.3)	447 (78.4)	487 (85.4)	547 (96.0)

BSRTC, Bethesda System for Reporting Thyroid Cytopathology; PTMC, papillary thyroid microcarcinoma; TIRADS, thyroid imaging reporting and data system.

### Cytopathological Examination and BRAF^V600E^ Mutation Analysis

Correlations of BSRTC categories with BRAF^V600E^ mutation and histopathological results were shown in **Table 3**. Only 33 (2.9%) of the thyroid nodules were diagnosed as non-diagnostic based on the FNA cytology; 12 (36.4%) of them were identified as PTMCs by postoperative histopathology. A total of 51 (42.1%) of the 121 nodules with indeterminate cytology were confirmed after surgery as PTMCs. Non-diagnostic and indeterminate cytology were found for 13.3% (154/1,157) of thyroid nodules, and the malignancy rates among these lesions were 40.9% (63/154). The malignancy rates in BSRTC I–VI lesions were 36.4%, 5.8%, 42.1%, 33.6%, 89.7%, and 98.1%, respectively. The rates of BRAF^V600E^ mutation in BSRTC I–VI lesions were 27.3%, 6.5%, 35.5%, 29.9%, 78.2%, and 87.0%, respectively. In BSRTC V–VI thyroid lesions, both the malignancy rates and the BRAF^V600E^ mutation rates increased obviously. The 28 nodules categorized as BSRTC V and four nodules categorized as BSRTC VI proved to be benign lesions postoperatively, including nodular goiter (n = 6), Hashimoto’s thyroiditis with fibrosis and calcification (n = 8), atypical adenoma with fibrosis, calcification, cystic degeneration or papillary hyperplasia (n = 14), subacute thyroiditis (n = 3), and atypical follicular epithelial hyperplasia (n = 1). The ROC curve demonstrated that the optimal cutoff point of cytologic results was BSRTC category V. The Az value, sensitivity, specificity, accuracy, PPV, and NPV were 0.910, 78.4%, 94.5%, 86.6%, 93.3%, and 81.9%, respectively.

**Table 3:** Correlations of BSRTC categories with BRAF^V600E^ mutation and histopathological results.

BSRTC categories	n	BRAF^V600E^ mutation	The rate of BRAF^V600E^ mutation	Malignant rate
I (Non-diagnostic) (n = 33)	9	Positive	27.3%	36.4%
	24	Negative		
II (Benign) (n = 417)	27	Positive	6.5%	5.8%
	390	Negative		
III (AUS/FLUS) (n = 121)	43	Positive	35.5%	42.1%
	78	Negative		
IV (FN/SFN) (n = 107)	32	Positive	29.9%	33.6%
	75	Negative		
V (Suspicious for malignancy) (n = 271)	212	Positive	78.2%	89.7%
	59	Negative		
VI (Malignancy) (n = 208)	181	Positive	87.0%	98.1%
	27	Negative		

AUS/FLUS, atypia undetermined significance/follicular lesion of undetermined significance; BSRTC, Bethesda System for Reporting Thyroid Cytopathology; FN/SFN, follicular neoplasm or suspicious for a follicular neoplasm.

Of the 1,157 thyroid nodules, 1,153 (99.7%) specimens had definite genetic results and the remaining four (0.3%) nodules showed suspicious positivity for BRAF^V600E^ mutation. BRAF^V600E^ mutation was detected in 487 (85.4%) of 570 PTMCs. Seventeen (2.9%) of the 587 benign thyroid nodules harbored the BRAF^V600E^ mutation. Of the 678 nodules with negative cytologic results, 111 (16.4%) nodules harbored the BRAF^V600E^ mutation, 100 (90.1%) of them were validated as PTMCs after surgery, while the remaining 11 (9.9%) nodules were diagnosed as benign lesions by surgery (n = 7) or F/U FNA and ultrasound (n = 4). The BRAF^V600E^ mutation rates were more frequent in the malignant category compared with the indeterminate category (BSRTC V *vs*. BSRTC III, *P* < 0.0001; BSRTC VI *vs*. BSRTC III, *P* < 0.0001) (**Table 3**). The Az value, sensitivity, specificity, accuracy, PPV, and NPV for BRAF^V600E^ mutation were 0.913, 85.4%, 97.1%, 91.4%, 96.6%, and 87.3%, respectively.

### Comparisons of TIRADS Classification, BSRTC, and BRAF^V600E^ Mutation

As shown in **Table 4**, sensitivity, specificity, accuracy, PPV, and NPV for BSRTC categories were significantly higher than those for TIRADS classification (*P* < 0.0001 for all comparisons). Moreover, BRAF^V600E^ mutation showed higher sensitivity, specificity, accuracy, PPV, and NPV than BSRTC categories (*P* = 0.002, *P* = 0.02, *P* < 0.0001, *P* = 0.017, and *P* = 0.006, respectively).

**Table 4:** Comparison of the diagnostic performance of different modalities for discrimination between benign and malignant thyroid nodules.

Diagnostic Modalities	A_z_*	Sensitivity (%)	Specificity (%)	Accuracy (%)	PPV (%)	NPV (%)
TIRADS	0.814 (0.791, 0.836)	69.1 (394/570)	85.2 (500/587)	77.3 (894/1157)	81.9 (394/481)	74.0 (500/676)
BSRTC	0.910 (0.892, 0.926)	78.4 (447/570)	94.5 (555/587)	86.6 (1002/1157)	93.3 (447/479)	81.9 (555/678)
BRAF^V600E^ mutation	0.913 (0.896, 0.929)	85.4 (487/570)	97.1 (570/587)	91.4 (1057/1157)	96.6 (487/504)	87.3 (570/653)
TIRADS + BSRTC	0.866 (0.845, 0.885)	91.9 (524/570)	81.3 (477/587)	86.5 (1001/1157)	82.9 (524/632)	91.2 (477/523)
BSRTC + BRAF^V600E^ mutation	0.943 (0.928, 0.956)	96.0 (547/570)	92.7 (544/587)	94.3 (1091/1157)	92.7 (547/590)	95.9 (544/567)
TIRADS + BSRTC + BRAF^V600E^ mutation	0.967 (0.955, 0.977)	92.1 (525/570)	96.6 (567/587)	94.4 (1092/1157)	96.3 (525/545)	92.6 (567/612)

*Numbers in parentheses are 95% confidence intervals.

BSRTC, Bethesda System for Reporting Thyroid Cytopathology; NPV, negative predictive value; PPV, positive predictive value; TIRADS, thyroid imaging reporting and data system.

Using TIRADS classification, BSRTC categories, and BRAF^V600E^ mutation as the criteria to distinguish malignant from benign thyroid nodules, the A_z_ value was 0.814 for TIRADS classification and 0.910 for BSRTC categories, and the A_z_ value for BRAF^V600E^ mutation was 0.913. The A_z_ values of both BSRTC and BRAF^V600E^ mutation analysis were significantly higher as compared with TIRADS classification (*P* < 0.0001 and *P* < 0.0001, respectively), whereas no statistically significant difference was found between them (*P* = 0.788).

### Diagnostic Value of the Combinations of Different Methods

**Figure 2:**
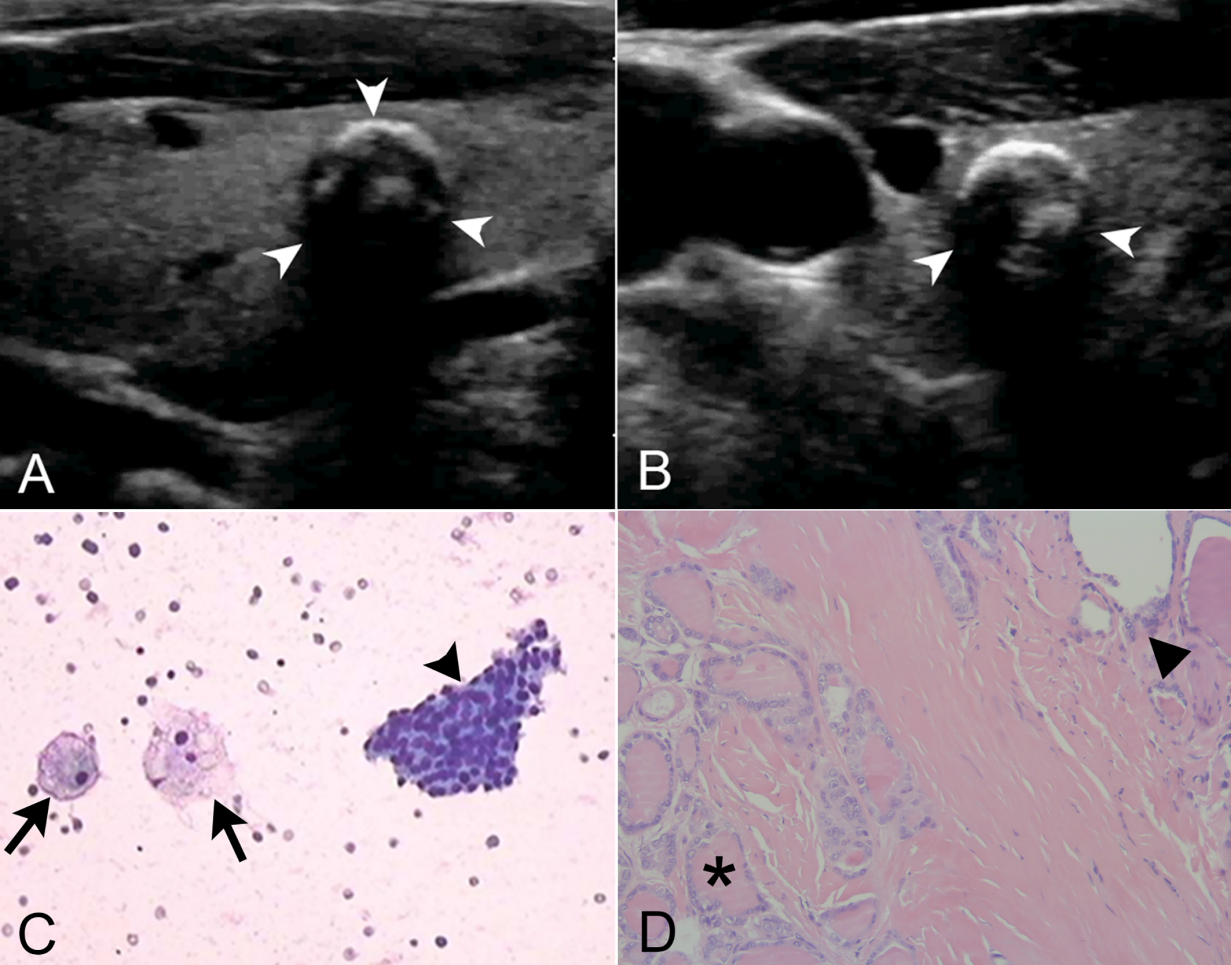
The combined use of thyroid imaging reporting and data system (TIRADS), Bethesda System for Reporting Thyroid Cytopathology (BSRTC), and BRAF^V600E^ mutation analysis in a 59-year-old man with 7.2 mm × 6.1 mm papillary thyroid microcarcinoma (PTMC). **(A)** Longitudinal ultrasound image showed a regularly shaped, indistinctly marginated, hypoechoic nodule with multiple macrocalcifications in the right lobe of the thyroid gland (arrow heads). **(B)** Transrectal ultrasound image more clearly showed the peripheral half ring-like macrocalcifications (arrow heads). **(C)** Cytological pathology showed nodular goiter with cystic change. Lamellar follicular epithelial cells (arrow head) and phagocytes (arrows) were seen, which was consistent with benign lesions (original magnification, ×20). **(D)** Tumor cells were seen at the lower left (asterisk), and normal thyroid follicles were at the upper right (triangle). Tumor cells were ground glass-like with large and crowded nuclei (original magnification, ×200). This nodule was classified as TIRADS 4a. The fine-needle aspiration (FNA) cytology result was categorized as BSRTC II, suggestive of a benign lesion. However, this nodule with negative cytologic result harbored the BRAF^V600E^ mutation and proven to be PTMC postoperatively. By the combined use of BRAF^V600E^ mutation analysis, this malignant thyroid nodule that was misdiagnosed as benign by ultrasound and cytology got a correct diagnosis.

The combined use of TIRADS and BSRTC significantly increased the sensitivity (91.9%) compared to cytology alone, while specificity (81.3%) and accuracy (86.5%) were not improved obviously. The combination of BSRTC and BRAF^V600E^ mutation analysis yielded a considerably high sensitivity, accuracy, and NPV than either BSRTC or BRAF^V600E^ mutation alone (*P* < 0.0001 for all comparisons). When the three methods were combined, accuracy increased to 94.4%, with an increased specificity and PPV to 96.6% and 96.3% (**Figure 2**). The combination of the three methods yielded a higher specificity and PPV (*P* < 0.0001 and *P* = 0.008, respectively), whereas sensitivity and NPV were lower than those of the combination of BSRTC and BRAF^V600E^ mutation (*P* < 0.0001 and *P* = 0.015, respectively). The combination of the three methods could not improve the diagnostic accuracy compared with the combination of BSRTC and BRAF^V600E^ mutation (*P* = 1) (**Table 4**).

**Figure 3:**
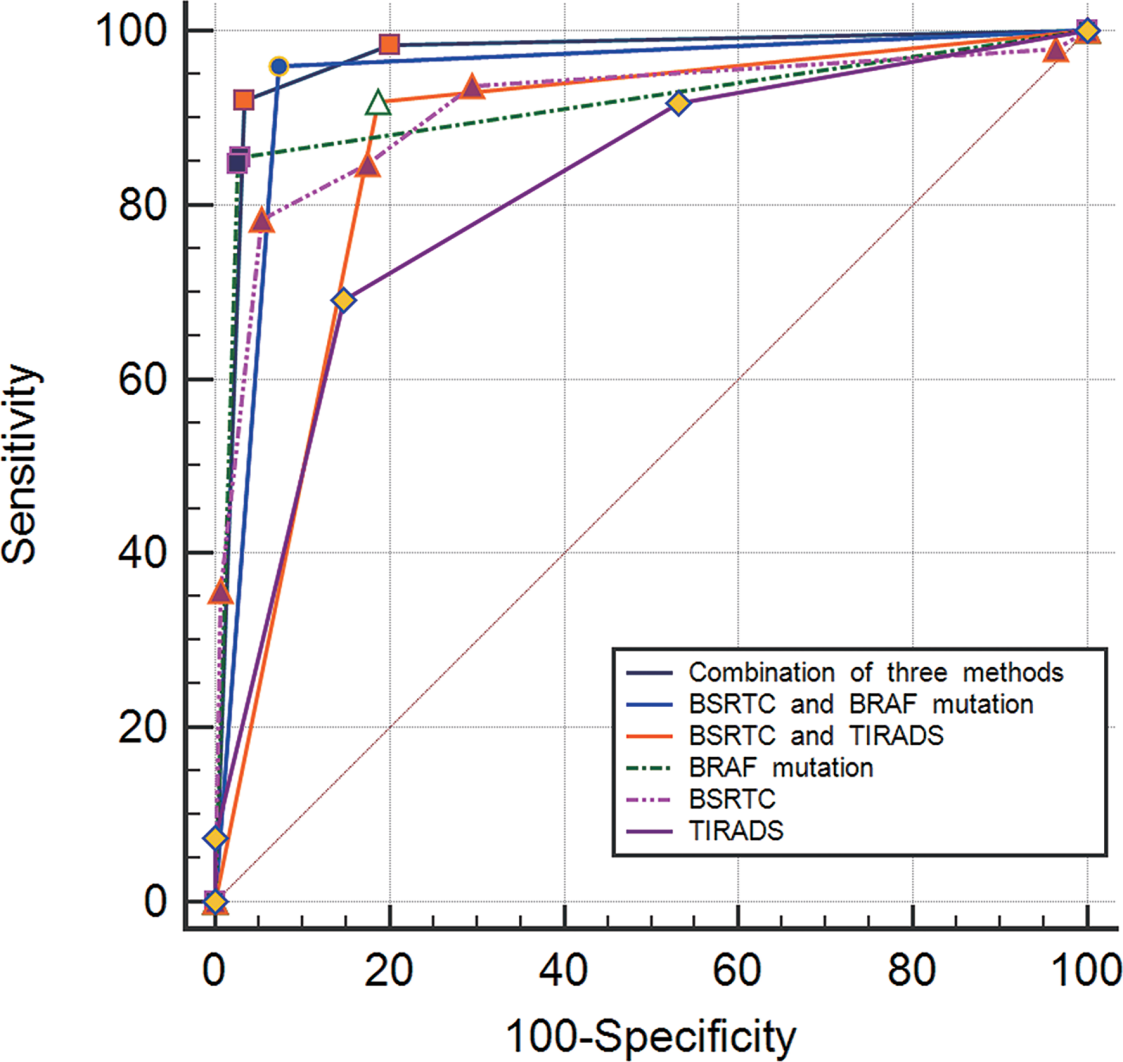
Graph of receiver operating characteristic (ROC) analyses of different diagnostic methods for distinguishing between benign and malignant thyroid nodules.

The A_z_ value for the combined use of the three methods was maximal and that for the combination of BSRTC and BRAF^V600E^ mutation analysis was slightly lower; a statistical difference was found between them (*P* < 0.0001). The A_z_ value of the combination of BSRTC and BRAF^V600E^ mutation analysis was significantly higher as compared with each method alone or the combination of TIRADS and BSRTC (TIRADS, *P* < 0.0001; BSRTC, *P* = 0.0001; BRAF^V600E^ mutation, *P* = 0.0001; TIRADS and BSRTC, *P* < 0.0001) (**Figure 3**).

### Relationship Between TIRADS Classification and BRAF^V600E^ Status for Thyroid Nodules With Indeterminate Cytology

Of 1,157 thyroid lesions, 121 (10.5%) nodules were classified as BSRTC III, 51 (42.1%) of them were malignant and 70 (57.9%) were benign. TIRADS classification and BRAF^V600E^ mutation status in 121 thyroid nodules with indeterminate cytology were shown in **Table 5**. The malignancy rates of nodules categorized as TIRADS 4a, 4b, 4c, and 5 were 11.6%, 42.9%, 76.5%, and 100%, respectively. BRAF^V600E^ mutation status was significantly associated with TIRADS classification. Except for only two nodules classified as TIRADS 5, BRAF^V600E^ mutation rate increased as the level of TIRADS classification increased. In nodules classified as TIRADS 4a, 4b, and 4c, the BRAF^V600E^ mutation rates were 11.6%, 38.1%, and 61.8%, respectively. Thyroid nodules with different TIRADS categories showed significant differences in the BRAF^V600E^ mutation rate (4a *vs*. 4b, *P* = 0.005; 4b *vs*. 4c, *P* = 0.04, respectively).

**Table 5:** TIRADS classification and BRAF mutation status in 121 thyroid nodules with indeterminate cytology.

TIRADS Classification	n	Malignancy (%)	BRAF^V600E^ mutation	
			Positive (%)	Negative (%)
4a	43	5 (11.6)	5 (11.6)	38 (88.4)
4b	42	18 (42.9)	16 (38.1)	26 (61.9)
4c	34	26 (76.5)	21 (61.8)	13 (38.2)
5	2	2 (100)	1 (50)	1 (50)
Total	121	51 (42.1)	43 (35.5)	78 (64.5)

TIRADS, thyroid imaging reporting and data system.

### Characteristics Analysis of PTMCs With BSRTC Category III

Of the 51 nodules that were classified as BSRTC III but proven to be PTMCs by histopathology, 10 (19.6%) and 32 (62.7%) were found to associated with Hashimoto’s thyroiditis and calcifications. No significant difference was found between malignant and benign nodules with respect to Hashimoto’s thyroiditis and calcifications (*P* = 0.133 and *P* = 0.278, respectively). The malignancy rates of thyroid nodules with BSRTC III increased as the level of TIRADS classification increased (*P* < 0.0001). Forty-one (80.4%) of 51 PTMCs were positive for BRAF^V600E^ mutation, indicating that BRAF^V600E^ mutation was very helpful for the detection of PTMCs with BSRTC III. Of the 51 PTMCs with indeterminate cytology, TIRADS categories were suggestive of malignancy in 28 (54.9%) and benign lesion in 23 (45.1%). The diagnostic accuracy of the molecular method in the 121 indeterminate nodules was 90.1% (109/121), which was significantly higher than that of TIRADS classification, 74.4% (90/121) (*P* = 0.002) (**Table 6**).

**Table 6:** Correlations of clinicopathologic and ultrasound findings and BRAF^V600E^ mutation with final diagnosis in BSRTC III category.

Clinicopathologic, ultrasound findings, and BRAF^V600E^ mutation	n (%)	BSRTC III		*P* value
		Malignant (n = 51)	Benign (n = 70)	
**Hashimoto’s thyroiditis**				
Present	17 (14.0)	10 (19.6)	7 (10.0)	0.133
Absent	104 (86.0)	41 (80.4)	63 (90.0)	
**Calcifications**				
Present	69 (57.0)	32 (62.7)	37 (52.9)	0.278
Absent	52 (43.0)	19 (37.3)	33 (47.1)	
**TIRADS classification**				
4a	43 (35.5)	5 (11.6)	38 (88.4)	<0.0001
4b	42 (34.7)	18 (42.9)	24 (57.1)	
4c	34 (28.1)	26 (76.5)	8 (23.5)	
5	2 (1.7)	2 (100.0)	0	
**BRAF^V600E^ mutation**				
Positive	43 (35.5)	41 (80.4)	2 (2.9)	<0.0001
Negative	78 (64.5)	10 (19.6)	68 (97.1)	

BSRTC, Bethesda System for Reporting Thyroid Cytopathology; TIRADS, thyroid imaging reporting and data system.

## Discussion

It has been reported that 30%–65% of patients with PTMC had central lymph node metastasis (detected only on pathology) (4). Our results suggested that lymph node metastases were found in 150 (28.5%) patients with PTMC, which was slightly lower than those in literature reports. However, in our study, only four (2.7%) patients with cervical lymph node metastasis were observed by preoperative ultrasound because they were usually small and were obscured by the overlying thyroid gland. Hashimoto’s thyroiditis was an independent risk factor for PTC in children and adolescents (26). Our results had also shown that the proportion of patients combined with Hashimoto’s thyroiditis was significantly higher in the PTMC group than in the benign thyroid disease group. Multifocality is another independent risk factor that could predict lymph node metastasis and recurrence (27). In our study, tumor multifocality was observed in 43 (8.2%) cases with PTMC.

Surgical treatment strategies, such as hemithyroidectomy or total thyroidectomy and whether to perform lymph node dissection, depend on the qualitative diagnosis of thyroid nodules, the degree of risk of nodules, lymph nodal involvement, or the presence of multiglandular disease or thyroiditis (28). In the treatment of differentiated thyroid cancer, in the absence of enlarged lymph nodes, the role of routine central lymph node dissection (RCLD) remains controversial. In more demolitive surgery, interventions are exposed to more severe complications, such as permanent hypoparathyroidism and unilateral temporary laryngeal nerve paralysis. RCLD for differentiated thyroid cancer is not recommended because a simple thyroidectomy not combined with RCLD is associated with a low locoregional recurrence rate (29). In all patients with incidental PTMCs, the surgical indication is given for symptomatic disease, for impairment of thyroid function, or for failure to respond to medical therapy or unable to continue, since incidental PTMCs have little biological aggressiveness and are susceptible to metabolic radioiodine therapy (30).

Risk stratification of thyroid malignancy by using the number of suspicious ultrasound features allows for a practical and convenient TIRADS. The observation indices of malignant thyroid nodules used in this study were more comprehensive and detailed, including more meaningful ultrasonographic features such as irregular thick halo, suspicious cervical lymph nodes, and findings for predicting malignant partially cystic thyroid nodules. In the present study, the malignancy risk increased as the level of TIRADS classification increased. The malignancy risks of categories 4a, 4b, 4c, and 5 were 14.6%, 36.4%, 80.2%, and 100%, respectively, which were almost exactly in agreement with the ideal range (4a: 2–10, 4b: 10–50, 4c: 50–95, 5: >95) of TIRADS classification proposed by Kwak et al. (23). This result demonstrated that the classification method used in this study and the interpretation of ultrasound images were accurate. The present study indicated that TIRADS classification had a better diagnostic performance for the differential diagnosis of thyroid nodules, and the Az value, sensitivity, specificity, and accuracy were 0.815, 69.1%, 85.2%, and 77.3%, respectively. A recent study reveals that TIRADS classification can be used as a clinical parameter for deciding the BRAF mutation test in thyroid nodules with AUS/FLUS cytology (31). A similar conclusion was also drawn from our present study because the BRAF^V600E^ mutation rate increased as the level of TIRADS classification increased in 121 thyroid nodules with indeterminate cytology and different TIRADS categories showed significant differences in the BRAF^V600E^ mutation rate.

Thyroid FNA cytology is a cost-effective procedure that provides specific diagnosis rapidly with minimal complications. It plays an important role in the determination of clinical treatment and management plan. A reliable definitive diagnosis of PTC is usually straightforward in fine-needle aspirates of conventional PTC whenever the characteristic papillary and/or flat honeycomb sheet-like architecture and the typical nuclear features of chromatin pallor, nuclear enlargement, crowding, grooves, and pseudoinclusions are encountered. Cytology could help distinguish conventional PTC and the different PTC subtypes, such as “noninvasive follicular thyroid neoplasm with papillary-like nuclear features” (NIFTP) and follicular variant of PTC (FVPTC) by observing cytomorphologic features (32). Different subtypes of PTC have different molecular features. Conventional PTC with BRAF^V600E^ mutations readily demonstrates nuclear features of PTC in cytology specimens as compared to RAS-driven tumors (NIFTP and FVPTC). At the molecular level, NIFTP shows a very high association with other follicular-pattern neoplasms, with RAS mutations being the most common, followed by PAX8/PPARγ translocations, THADA fusions, and BRAFK601E mutations (33–36). In contrast, BRAF^V600E^ mutations and RET fusions (common in conventional PTC) are absent in NIFTP. Different genetic characteristics of PTC subtypes also explain why some PTMCs in our study fail to detect BRAF mutations.

The BSRTC established a standardized category-based reporting system for thyroid FNA specimens and has proven to be an effective and robust thyroid FNA classification scheme to guide the clinical treatment. Lin et al. (11) has shown that the proportion of non-diagnostic and indeterminate samples accounted for 30%. However, the results of our study indicated that non-diagnostic and indeterminate cytology was found for 13.3% (154/1157) of thyroid nodules, which was much lower than that of the previous report. This finding may be due to years of experience of pathologists and their training in cytopathology or the proficiency of the FNA operators. A recent study of Cibas and Ali (25) has shown that the rates of malignancy in BSRTC I–VI were 5%–10%, 0%–3%, 10%–30%, 25%–40%, 50%–75%, and 97%–99%, respectively. The rates of malignancy in nodules that were classified as BSRTC I, III, and V in this present study were significantly higher than the recommended range, whereas the rates of malignancy in BSRTC II, IV, and VI thyroid nodules were close to the ideal range. The malignancy rates among BSRTC I and III lesions were as high as 40.9%, indicating a relatively conservative attitude of the pathologist for interpreting the cytology. In our study, the high risk of malignancy associated with AUS/FLUS cases was even higher than cases classified as Bethesda IV (42.1% *vs.* 33.6%). Therefore, it was more important to further combine genetic testing methods to improve the detection rates for PTMCs with BSRTC III.

The mutation rate of BRAF^V600E^ in PTMCs in this study population was 85.4%, which is close to the Korea population with 52%–83% prevalence of BRAF mutation (37). The rates of BRAF gene mutation in Chinese and Korea patients are significantly higher than those in western populations (USA and Europe) with the consistent prevalence of 40%–45% (38). Furthermore, this difference in prevalence might also be due to the different methods and techniques used for gene mutation testing. In this study, polymerase chain reaction (PCR)-based method amplification refractory mutation system (ARMS) was used for BRAF^V600E^ mutation test but not traditional DNA sequencing. The ARMS assays could maximize the number of samples and improve both the sensitivity and speed of analysis compared with DNA sequencing (39). In our study, the specificity (97.1%) of BRAF^V600E^ mutation was slightly lower than the result of the meta-analysis (pooled specificity 99.0%) by Jia et al. (37). This finding may be related to the oversensitivity of ARMS method so that the BRAF^V600E^ mutation has been detected in a small number of benign nodules. BRAF^V600E^ mutation analysis could also significantly increase the detection rates of FNA for PTMCs with various levels of TIRADS classification, especially those lesions classified as TIRADS 4.

The prevalence of BRAF^V600E^ mutation (80.4%) among cytologically indeterminate samples in our study was significantly higher compared with that recently reported by Su et al. (9), who found a 40.1% incidence in BSRTC III nodules. The result of our study indicated that BRAF^V600E^ status would be helpful for diagnosing the status of nodules with indeterminate cytology, which was consistent with those of some previous studies (13, 40) but conflicted with the findings of other researchers (15–17), who had reported that BRAF mutation analysis on an FNA specimen with indeterminate cytology might not be as helpful as indicated by previous studies (13, 40). Our data suggested that both BRAF^V600E^ mutation analysis and TIRADS classification were potentially useful adjuncts to evaluate thyroid nodules that were difficult to diagnose. Moreover, the diagnostic accuracy of the molecular method for the indeterminate nodules was significantly higher than that of TIRADS classification. The application of BRAF^V600E^ mutation analysis in FNA specimens classified as BSRTC III was more effective for thyroid nodules with higher TIRADS classification as compared with those nodules with lower TIRADS classification, demonstrating that TIRADS classification could be used to select patients for molecular analysis. Hashimoto’s thyroiditis and calcifications might be some factors that influence the differential diagnosis of benign and malignant thyroid nodules classified as BSRTC III. However, in our study, no significant relationship was observed between malignant and benign nodules with respect to Hashimoto’s thyroiditis and calcifications.

We found that BRAF^V600E^ mutation detection could significantly increase the sensitivity, accuracy, and NPV when combined with BSRTC. Of all the methods, the combined use of the three methods produced the best diagnostic performance, which was significantly higher than that for the combination of BSRTC and BRAF^V600E^ mutation analysis. However, Zhang et al. (20) has reported that the diagnostic performance of BSRTC combined with BRAF^V600E^ mutation analysis for differentiating high-risk thyroid nodules is significantly higher as compared with the combination of the three methods, which is not consistent with the result of our study. This finding, in part, might be due to the fact that investigators at different institutions use different criteria for the combined use of the three methods. In our study, the criteria for the combined use of the three methods were developed when considering the diagnostic performances of different methods and different combined situations that BRAF^V600E^ mutation, BSRTC, or TIARDS classification was suggestive of malignancy or benign possibility. This combined criteria allowed for a high sensitivity while improving specificity and accuracy for the differentiation of thyroid lesions. However, in the previous study of Zhang et al. (20), a nodule was considered positive as long as either method was positive when three diagnostic methods were used in combination. Such a combined criteria would inevitably lead to a certain degree increase in sensitivity, whereas the specificity was significantly reduced and the accuracy was not improved obviously.

This study had several limitations. Firstly, there was a selection bias because we excluded thyroid nodules without further histopathological diagnosis or F/U FNA and ultrasound to evaluate diagnostic performance. Secondly, not all thyroid nodules obtained histopathological results. Some nodules were classified as benign based on cytological diagnosis, F/U ultrasound, and BRAF^V600E^ gene mutation test, which might cause some false negative results and overestimate the sensitivity of BRAF^V600E^ mutation analysis. Thirdly, only high-risk thyroid nodules classified as TIRADS categories 4 and 5 were included in our study, so that the rate of malignant lesions was higher as compared to the general population with thyroid nodules.

## Conclusions

In summary, BRAF^V600E^ mutation analysis has the best diagnostic performance among the three methods. The combined use of ultrasound, cytology, and BRAF^V600E^ mutation analysis is the most efficient and objective method for diagnosing PTMC. Both BRAF^V600E^ mutation analysis and TIRADS classification are potentially useful adjuncts to differentiate thyroid nodules, especially indeterminate samples classified as BSRTC III, and select malignancy to guide the surgery.

## Data Availability Statement

The original contributions presented in the study are included in the article/**Supplementary Material**. Further inquiries can be directed to the corresponding author.

## Ethics Statement

The studies involving human participants were reviewed and approved by the Ethics Committee of Renji Hospital. The patients/participants provided their written informed consent to participate in this study.

## Author Contributions

Study concept and study design, JD, RH, and FL. Data acquisition, analysis, and interpretation, JD and RH. Article drafting or article revision for important intellectual content, all authors. Literature research, JD, RH, CC, and XM. Clinical studies, YS and JC. Statistical analysis, JD and RH. All authors contributed to the article and approved the submitted version.

## Funding

This study was supported by the Health and Family Planning Joint Research Project of Shanghai Pudong New Area Health Commission (grant number PW2019D-6) and the National Natural Science Foundation of China (grant numbers 82071954 and 81102014).

## Conflict of Interest

The authors declare that the research was conducted in the absence of any commercial or financial relationships that could be construed as a potential conflict of interest.

## Publisher’s Note

All claims expressed in this article are solely those of the authors and do not necessarily represent those of their affiliated organizations, or those of the publisher, the editors and the reviewers. Any product that may be evaluated in this article, or claim that may be made by its manufacturer, is not guaranteed or endorsed by the publisher.
