# Supplementary figures and images for: Diagnostic Efficacy of Ultrasound, Cytology, and BRAFV600E Mutation Analysis and Their Combined Use in Thyroid Nodule Screening for Papillary Thyroid Microcarcinoma
Source: Front Oncol. 2022 Jan 3;11:746776. doi: 10.3389/fonc.2021.746776 (PMC8761628; doi:10.3389/fonc.2021.746776)

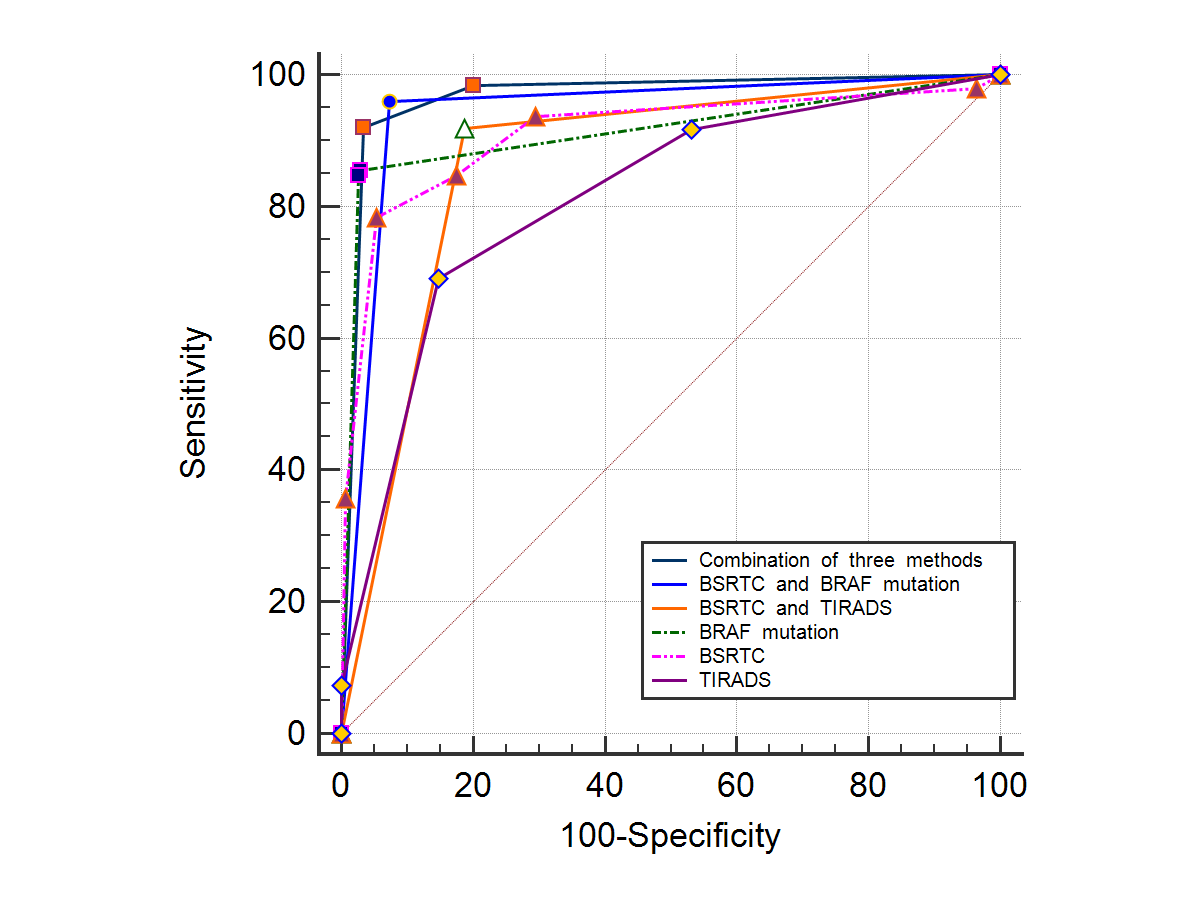

Supplement: Supplementary file 1 [file DataSheet_1.zip › Original Data and Statistical Analysis/Multiple comparison ROC curve-final.tif]

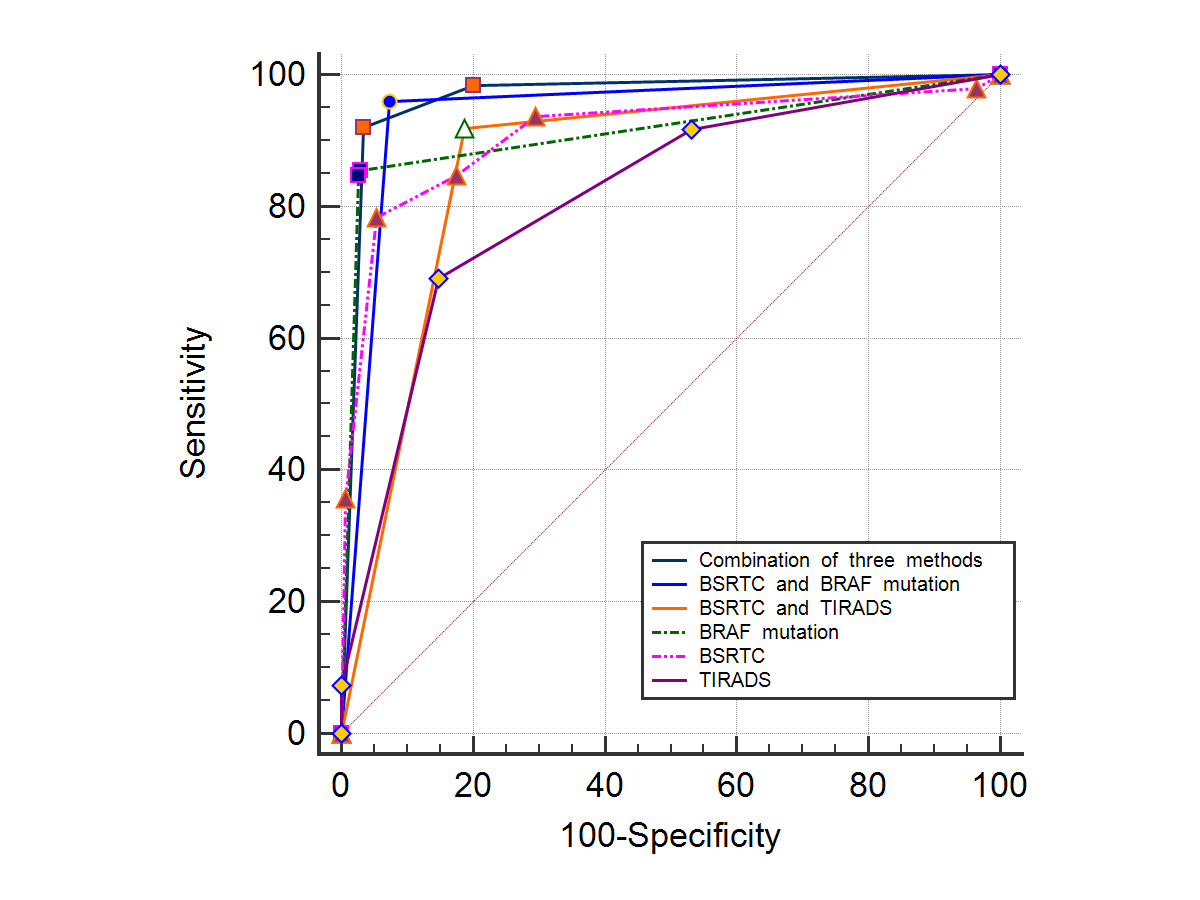

Supplement: Supplementary file 1 [file DataSheet_1.zip › Original Data and Statistical Analysis/Multiple comparison ROC curve.jpg]

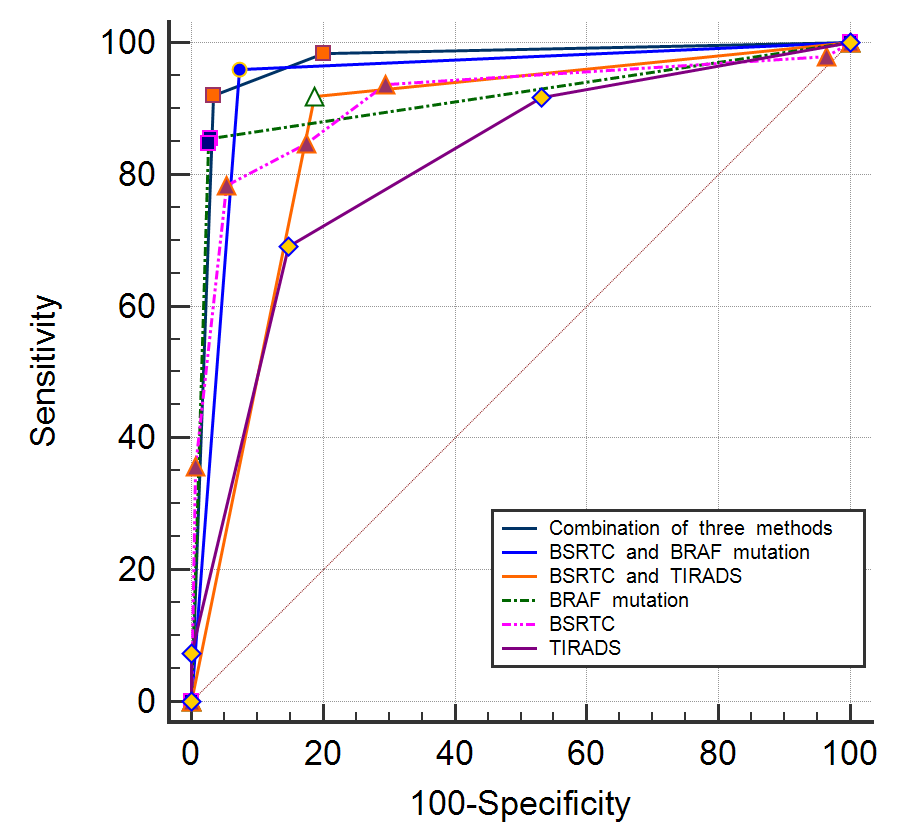

Supplement: Supplementary file 1 [file DataSheet_1.zip › Original Data and Statistical Analysis/Multiple comparison ROC curve.tif]
